# Alterations of brain local functional connectivity in amnestic mild cognitive impairment

**DOI:** 10.1186/s40035-018-0134-8

**Published:** 2018-11-07

**Authors:** Dan Zhen, Wei Xia, Zhong Quan Yi, Pan Wen Zhao, Jian Guo Zhong, Hai Cun Shi, Hua Liang Li, Zhen Yu Dai, Ping Lei Pan

**Affiliations:** 1 School of Nursing, Jiangsu Vocational College of Medicine, Yancheng, People’s Republic of China; 2 Department of Neurology, Affiliated Yancheng Hospital, School of Medicine, Southeast University, West Xindu Road 2#, Yancheng, Jiangsu Province, 224001 People’s Republic of China; 3 Department of Central Laboratory, Affiliated Yancheng Hospital, School of Medicine, Southeast University, West Xindu Road 2#, Yancheng, Jiangsu Province, 224001 People’s Republic of China; 4 Department of Radiology, Affiliated Yancheng Hospital, School of Medicine, Southeast University, West Xindu Road 2#, Yancheng, Jiangsu Province, 224001 People’s Republic of China

**Keywords:** Amnestic mild cognitive impairment, Default mode network, Meta-analysis, Regional homogeneity, Resting-state functional magnetic resonance imaging, Seed-based *d* mapping

## Abstract

**Background:**

Resting-state functional magnetic resonance imaging studies using a regional homogeneity (ReHo) method have reported that amnestic mild cognitive impairment (aMCI) was associated with abnormalities in local functional connectivity. However, their results were not conclusive.

**Methods:**

Seed-based *d* Mapping was used to conduct a coordinate-based meta-analysis to identify consistent ReHo alterations in aMCI.

**Results:**

We identified 10 studies with 11 datasets suitable for inclusion, including 378 patients with aMCI and 435 healthy controls. This meta-analysis identified significant ReHo alterations in patients with aMCI relative to healthy controls, mainly within the default mode network (DMN) (bilateral posterior cingulate cortex [PCC], right angular gyrus, bilateral middle temporal gyri, and left parahippocampal gyrus/hippocampus), executive control network (right superior parietal lobule and dorsolateral prefrontal cortex), visual network (right lingual gyrus and left middle occipital gyrus), and sensorimotor network (right paracentral lobule/supplementary motor area, right postcentral gyrus and left posterior insula). Significant heterogeneity of ReHo alterations in the bilateral PCC, left parahippocampal gyrus/hippocampus, and right superior parietal lobule/angular gyrus was observed. Exploratory meta-regression analyses indicated that general cognitive function, gender distribution, age, and education level partially contributed to this heterogeneity.

**Conclusions:**

This study provides provisional evidence that aMCI is associated with abnormal ReHo within the DMN, executive control network, visual network, and sensorimotor network. These local functional connectivity alterations suggest coexistence of functional deficits and compensation in these networks. These findings contribute to the modeling of brain functional connectomes and to a better understanding of the neural substrates of aMCI. Confounding factors merit much attention and warrant future investigations.

**Electronic supplementary material:**

The online version of this article (10.1186/s40035-018-0134-8) contains supplementary material, which is available to authorized users.

## Background

Amnestic mild cognitive impairment (aMCI) is a syndrome with subjects showing memory complaints and deficits but with normal activities of daily living [1]. Individuals with aMCI are at a high risk for developing Alzheimer’s dementia (AD), which is the most common type of dementia in the elderly and has been a growing public health problem worldwide [2, 3]. Understanding the neurobiology of aMCI may therefore have implications for early diagnosis and preventive interventions of AD.

Resting-state functional magnetic resonance imaging (rs-fMRI) is a promising imaging technique to investigate regional neural activity and large-scale brain networks [4, 5]. Rs-fMRI allows for exploring complex cognitive processes in vivo without application of a task and has been extensively used to study the pathophysiology of AD and aMCI [4, 6, 7]. Regional homogeneity (ReHo) is a reliable rs-fMRI analytic algorithm to explore local functional connectivity that measures the similarity of the resting state time series between one given voxel and its neighbor voxels [8, 9]. Over the past decade, many rs-fMRI studies have reported aberrant ReHo in patients with aMCI relative to healthy controls. ReHo can be applied to distinguish patients with aMCI from healthy controls with an accuracy of 90.32% (sensitivity 86.21% and specificity 93.94%) by employing a support vector machine-based approach [10]. In addition, ReHo alterations have been observed to correlate with cognitive and memory impairment in patients with aMCI [11–14]. However, despite considerable progress in the better understanding of aMCI pathophysiology, the findings from ReHo studies have been less consistent than expected. For example, ReHo alterations in the posterior cingulate cortex (PCC)/precuneus in patients with aMCI relative to healthy controls have been controversial. Decreased ReHo, increased ReHo, or null findings in this region have been reported. Thus, it is of keen interest to overcome such inconsistency and characterize ReHo alterations in patients with aMCI by quantitatively pooling these studies. However, no such study has so far been conducted.

Our primary objective of the present study was to identify the consistent ReHo alterations in aMCI across studies via a meta-analytic approach. Seed-based *d* Mapping (SDM), a fully-validated technique for coordinate-based meta-analysis of neuroimaging studies [15–17], was utilized to quantitatively synthesis the whole-brain ReHo results in a voxel-wise manner. Additionally, we aimed to conduct meta-regression analyses to explore the potential impact of relevant demographic and clinical variables on ReHo changes in patients with aMCI.

## Methods

### Literature search and study selection

The electronic databases of PubMed, Web of Science, and Embase were systematically searched up until 14 July, 2017, using the following keywords and combinations: (“mild cognitive impairment” or “mci”) AND (“regional homogeneity” or “ReHo” or “local connectivity”). Reference lists from relevant studies were further reviewed to detect additional suitable articles. The search was updated on 8 May, 2018.

Study selection was according to the following inclusion criteria: 1) Patients enrollment met the diagnostic criteria for aMCI; 2) the study employed rs-fMRI to measure ReHo differences between patients with aMCI and matched healthy controls; 3) the study utilized a voxel-based statistical analysis at the whole-brain level; 4) three-dimensional stereotactic coordinates in either Talairach or Montreal Neurological Institute (MNI) space were explicitly reported; and 5) the study was an original article that was peer-reviewed and published in an English-language journal. Studies were excluded if they enrolled patients with non-aMCI. Studies were also excluded if they limited their analyses to specific regions of interest (ROIs), or used two-dimensional analyses, or did not report peak stereotactic coordinates. Only one study was selected in the final meta-analysis in case that two or more published studies had the same first author and data acquisition methods, and similar patient demographic characteristics, clinical variables, data analysis, and imaging results. Only pre-treatment or baseline data were included in case of longitudinal studies.

We used a 20-point checklist (Additional file 1), which was based on previous neuroimaging meta-analyses to evaluate the quality of each eligible study. Our study was performed in accordance with the Meta-analysis of Observational Studies in Epidemiology (MOOSE) guidelines [18].

### Data analysis

#### Main voxel-wise meta-analysis by pooling all included studies

The current version 5.15 of the SDM software package (www.sdmproject.com) was used to test for significant spatial convergence of ReHo findings across studies in patients with aMCI relative to healthy controls. The SDM approaches have been widely applied in neuropsychiatric disorders and the processes have been described in detail elsewhere [15, 19–25]. Peak three-dimensional stereotactic coordinates and their effect sizes of ReHo differences between patients with aMCI and healthy controls were firstly extracted from each study [19, 22]. A standard MNI map of ReHo changes for each study was then separately recreated with an un-normalised anisotropic Gaussian kernel (full width at half maximum [FWHM] = 20 mm) [19, 22, 23]. Of note, this Gaussian kernel is not used to smooth any image but to assign indicators of proximity to reported coordinates [20]. The mean map was further generated by voxel-wise calculation of the mean of the study maps. A random-effect model was applied, which takes into account the sample size, intra-study variability, and between-study heterogeneity [19, 22, 23]. The following thresholds were used to obtain the final SDM statistical map: uncorrected *p* < 0.005, peak height Z ≥ 1, and cluster extent ≥20 voxels. It should be noted that this uncorrected significance has been found in SDM method to be approximately equivalent to a corrected *p*-value = 0.05 [19, 22, 23].

### Reliability analysis

To test the reliability of the brain areas identified in the main voxel-wise meta-analysis, we performed whole-brain voxel-based jackknife sensitivity analyses by repeating the same analyses with the consecutive exclusion of each study [19, 20, 23, 24].

### Heterogeneity analysis

For assessing between-study heterogeneity of individual clusters, a random-effect model with Q statistics was utilized. *P* < 0.005, peak height z ≥ 1, and cluster extent ≥20 voxels were considered significant [26].

### Publication bias analysis

Possible publication bias was assessed with an Egger test by extracting the values from the relevant peaks in the main voxel-wise meta-analysis. A threshold at *p* < 0.05 indicated statistical significance.

### Subgroup meta-analysis

Subgroup meta-analyses of studies using 3.0 T MRI scanners (*N* = 9) and of studies that matched for sex between patients with aMCI and healthy controls (*N* = 10) were conducted to investigate the possible effects of the results on the overall conclusions.

### Meta-regression analysis

A meta-regression analysis was conducted to assess the severity of general cognitive function impairment examined by MMSE scores that correlated with the ReHo alterations in the patient samples. We also investigated the influence of demographic factors, such as gender distribution (female ratio), age, and educational level, on the ReHo changes in the aMCI samples. These analyses were performed with simple linear regression models. A stringent statistical threshold at *p* < 0.0005 and a cluster extent of 20 voxels were used [19, 21].

## Results

### Literature search and study selection

**Fig. 1:**
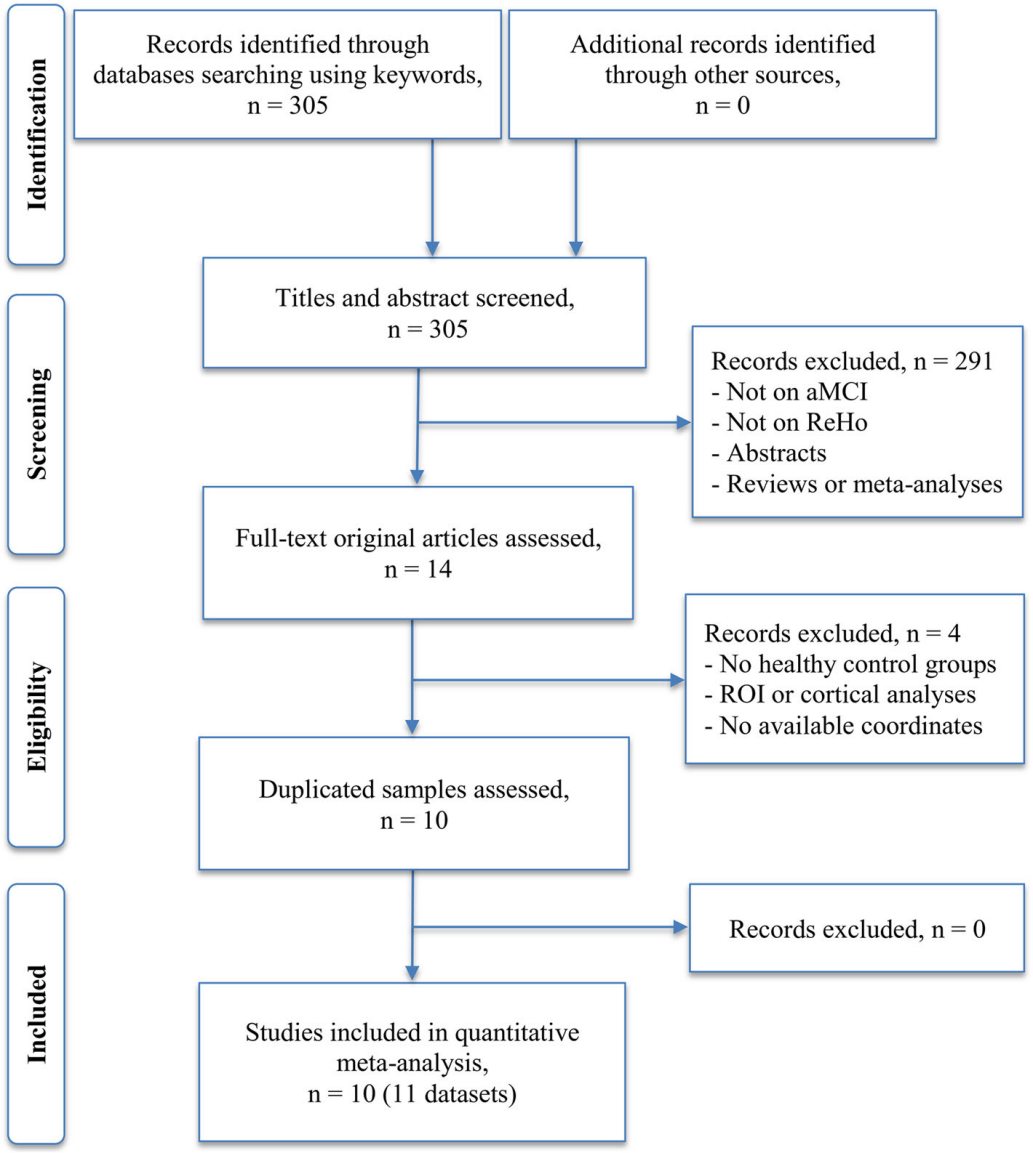
Flow chart for the literature selection. Abbreviations: aMCI, amnestic mild cognitive impairment; ReHo, regional homogeneity; ROI, region of interest

The systematic literature search yielded 305 titles and abstracts. Of these results, we identified 10 studies with 11 datasets suitable for final inclusion (378 patients, 435 controls) [10, 12–14, 27–32]. The detailed study selection process for the meta-analysis is shown in the flowchart Fig. 1. No significant differences were observed between the aMCI samples and HC groups regarding mean age (standardized mean difference [SMD] = 0.061; 95% confidence interval [CI] = − 0.096 to 0.218, z = 0.76, *p* = 0.45) or education level (SMD = − 0.26; 95% CI = − 0.57 to 0.037, z = 1.72, *p* = 0.086). However, an unbalance of gender distribution between the aMCI samples and HC groups (relative risk = 1.22, 95% CI = 1.05 to 1.40, z = 2.69, *p* = 0.007) was observed. All the studies included except for the study by Cha [32] matched for sex. In addition, the aMCI groups had significantly lower mean MMSE scores than that of the HC groups (SMD = − 1.70; 95% CI = − 2.26 to − 1.15, z = 6.00, *p* = 0.000). The quality of each included study was acceptable, with the quality score no less than 18 (a maximum score = 20). The demographic, clinical, and technical characteristics as well as the quality score of each eligible study are summarized in Table 1.

**Table 1:** Summary of ReHo studies included in the meta-analysis

Study	Sample (female)		Mean Age (SD)	MMSE (SD)	Education (SD)	Scanner strength	Software	FWHM	Threshold	Quality score^c^
Bai et al., 2008 [31]	aMCI	20 (10)	71.3 (3.8)	27.2 (1.6)	14.0 (3.1)	GE 1.5 T	SPM2, REST	4 mm	0.005, uncorrected	19
	HC	20 (11)	69.4 (3.8)	28.3 (1.4)	13.8 (4.0)					
Zhang et al., 2010 [11]	aMCI	48 (18)	72.04 (4.42)	27 (26–28)^d^	15.5 (12–16)^d^	GE 1.5 T	SPM2, REST	4 mm	0.05, corrected	20
	HC	36 (19)	71.64 (3.72)	29 (27–29)^d^	16 (12–16)^d^					
Liu et al., 2014 [17]	aMCI	12 (11)	59.3 (3.3)	26.4 (0.9)	10.5 (1.81)	GE 3.0 T	SPM5, REST	4 mm	0.01, corrected	20
	HC	12 (8)	60.6 (5.8)	29.8 (0.4)	10.6 (2.06)					
Cha et al., 2015 [32]	aMCI	34 (16)	68.5 (8.0)	27.1 (2.1)	11.5 (5.2)	Philips 3.0 T	AFNI	6 mm	0.05 corrected	20
	HC	62 (45)	68.4 (7.9)	28.6 (1.9)	10.9 (5.2)					
Wang et al., 2015 [29]	aMCI	30 (12)	69.1 (5.8)	26.2 (2.2)	NA	Philips 3.0 T	SPM8, BRAT	6 mm	0.01, uncorrected	18
	HC	32 (17)	70.1 (5.5)	28.1 (1.5)	NA					
Ni et al., 2016 [14]	aMCI	26 (14)	71 (9)	25 (1.48)	12 (3.08)	Philips 3.0 T	SPM8, REST, DPARSFA	6 mm	0.05, corrected	20
	HC	28 (11)	70 (9)	29 (1.09)	15 (1.41)					
Long et al., 2016 [10]	aMCI	29 (16)	66.55 (8.36)	23.38 (3.03)	10.14 (4.51)	Siemens 3.0 T	SPM8, REST	4 mm	0.01, corrected	20
	HC	33 (21)	62.91 (8.08)	27.94 (1.60)	11.00 (4.12)					
Yuan et al., 2016 [13]	aMCI	79 (37)	68.16 (6.67)	26.21 (2.69)	12.43 (3.01)	Siemens 3.0 T	SPM8, REST, DPARSFA	8 mm	0.01, corrected	20
	HC	119 (62)	69.65 (7.60)	28.21 (1.46)	11.84 (3.21)					
Yuan et al., 2016 [13]	aMCI	36 (19)	66.8 (9.5)	24.9 (3.4)	10.0 (4.1)	Siemens 3.0 T	SPM8, DPARSF	4 mm	0.01, corrected	20
	HC	46 (27)	64.3 (7.8)	28.5 (2.0)	11.4 (5.1)					
Luo et al., 2018 [28]	aMCI^a^	32 (15)	72.43 (4.25)	28.34 (1.68)	16.47 (2.24)	Philips 3.0 T	SPM8, REST, DPARSF	6 mm	0.01, corrected	20
	aMCI^b^	32 (15)	74.90 (5.27)	27.16 (1.71)	15.25 (2.65)					
	HC	49 (31)	73.33 (4.60)	29.02 (1.20)	16.24 (2.60)					

Abbreviations: *ReHo* Regional Homogeneity, *aMCI* amnestic Mild Cognitive Impairment, *SD* Standard Deviation, *MMSE* Mini-Mental State Examination, *FWHM* Full Width at Half Maximum, *SPM* Statistical Parametric Mapping, *REST* the Resting-State fMRI Data Analysis Toolkit, *DPARSF* Data Processing Assistant for Resting-State fMRI software, *DPARSFA* DAPRSF Advanced edition, *AFNI* Analysis of Functional NeuroImages, *BRAT* Brainnetome fMRI toolkit, *NA* Not Available, ^a^single-domain aMCI; ^b^multi-domain aMCI; ^c^a maximum score of 20 for each study; ^d^reported with median and interquartile range

### ReHo differences between patients with aMCI and healthy controls

**Fig. 2:**
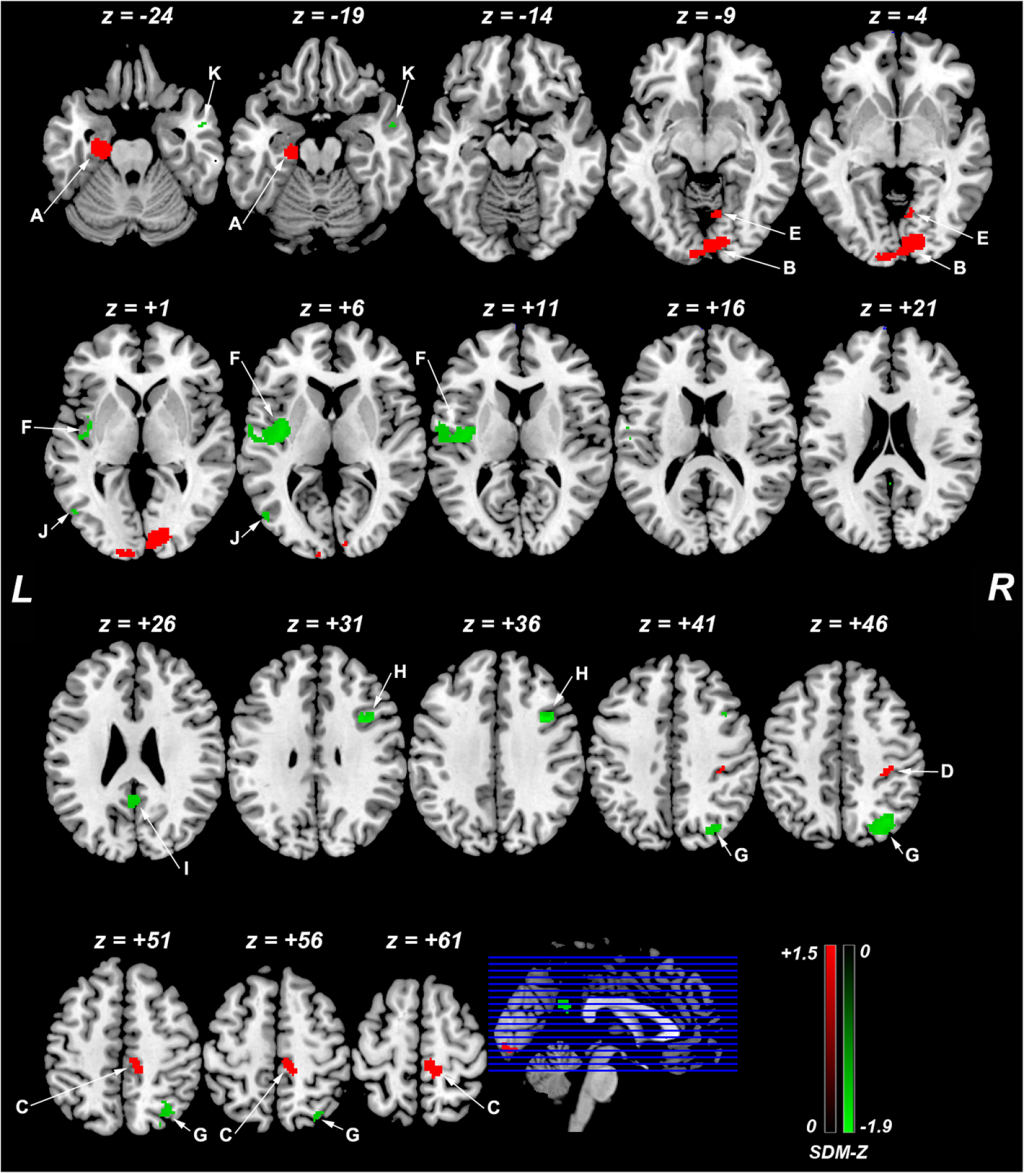
ReHo differences in patients with aMCI and healthy controls from the main voxel-wise meta-analysis. Abbreviations: ReHo, regional homogeneity; aMCI, amnestic mild cognitive impairment; L, left; R, right; SDM, Seed-based *d* Mapping; **a**, left parahippocampal gyrus (extending to the left hippocampus); **b**, right lingual gyrus (extending to the left middle occipital gyrus); **c**, right paracentral lobule (extending to the supplementary motor area); **d**, postcentral gyrus; **e**, right lingual gyrus; **f**, left insula, **g**, right superior parietal lobule (extending to the right angular gyrus); **h**, right dorsolateral prefrontal cortex; **i**, bilateral posterior cingulate cortex, **j**, left middle temporal gyrus, **k**, right left middle temporal gyrus. The color bar (increases of ReHo in red and decreases in green) indicates the maximum and the minimum SDM-Z values

The voxel-wise SDM results showed increased ReHo in the left parahippocampal gyrus (extending to the left hippocampus), right lingual gyrus (extending to the left middle occipital gyrus), right paracentral lobule (extending to the supplementary motor area), and postcentral gyrus, and decreased ReHo in the left insula, right superior parietal lobule (extending to the right angular gyrus), right dorsolateral prefrontal cortex (DLPFC), bilateral PCC, and bilateral middle temporal gyri in patients with aMCI compared to healthy controls. Details regarding the location, size, and peak density of each cluster are presented in Table 2. The SDM map is illustrated in Fig. 2.

**Table 2:** ReHo alterations in patients with aMCI relative to healthy controls

	Anatomical label	Peak MNI coordinate (x, y, z)	Voxels	SDM-Z value	*p* value (SDM)	Sensitivity analysis	*p* value (Egger’s test)
Increased ReHo	A. Left parahippocampal gyrus/hippocampus (BAs 28 and 35)	−22, −20, −22	197	1.5	0.0003	10/11	0.03
	B. Right lingual gyrus/left middle occipital gyrus (BAs 17 and 18)	10, −88, −6	488	1.4	0.0005	10/11	0.8
	C. Right paracentral lobule/supplementary motor area (BAs 6 and 4)	10, −30, 54	190	1.5	0.0003	9/11	0.6
	D. Right postcentral gyrus (BA 3)	34, −28, 44	45	1.3	0.001	9/11	0.8
	E. Right lingual gyrus (BA 18) G	10, −66, − 6	38	1.0	0.004	9/11	0.9
Decreased ReHo	F. Left insula (BA13)	−38, −10, 8	525	−1.8	0.001	9/11	0.9
	G. Right superior parietal lobule/angular gyrus (BAs 7 and 39)	34, −72, 42	306	−1.9	0.0007	11/11	0.9
	H. Right dorsolateral prefrontal cortex (BA 9)	34, 10, 36	94	−1.9	0.0005	9/11	0.2
	I. Right/Left posterior cingulate gyrus (BAs 23 and 30)	2, −48, 24	67	−1.7	0.002	7/11	0.01
	J. Left middle temporal gyrus (BA 37)	−50, −70, 0	22	−1.6	0.003	9/11	1.0
	K. Right middle temporal gyrus (BA 21)	54, 2, −20	16	−1.6	0.004	9/11	0.9

Abbreviations: *ReHo* Regional Homogeneity, *aMCI* amnestic Mild Cognitive Impairment, *MNI* Montreal Neurological Institute, *SDM* Seed-based *d* Mapping, *BA* Brodmann area

### Reliability analysis

The sensitivity analyses revealed that most of the areas found in the SDM map had very high reliability, with areas replicable in at least 9 out of 11 combinations of datasets. Decreased ReHo in the bilateral PCC was less robust than other areas, replicable in 7 out of 11 combinations of datasets (Table 2).

### Heterogeneity analysis

**Fig. 3:**
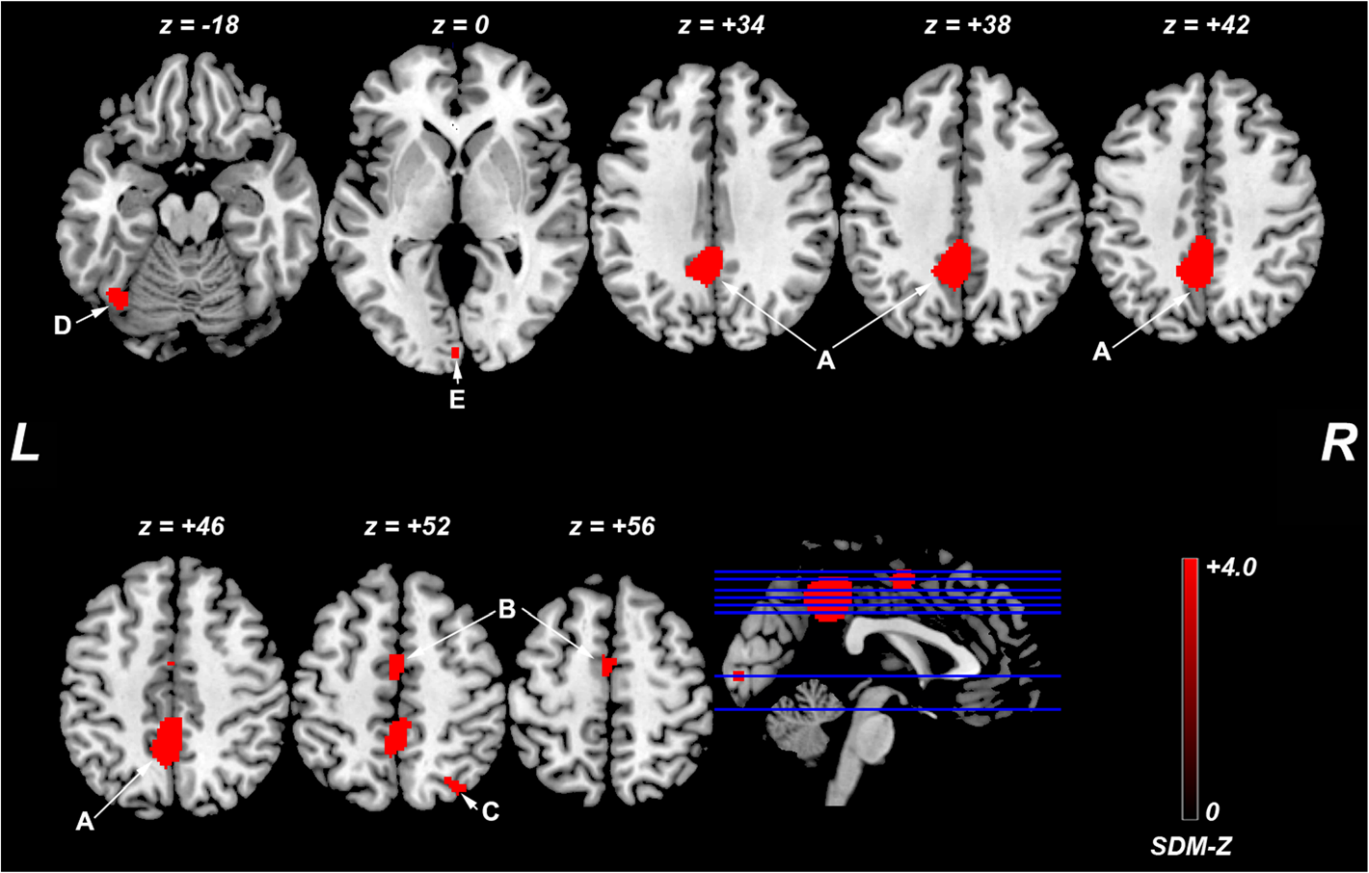
Regions with ReHo heterogeneity across studies. Abbreviations: SDM, Seed-based *d* Mapping; L, left; R, right; **a**, bilateral posterior cingulate cortex (extending to the precuneus); **b**, left supplementary motor area, **c**, right superior parietal lobule (extending to the angular gyrus); **d**, left fusiform gyrus, **e**, left lingual gyrus. The color bar indicates the maximum and the minimum SDM-Z values

Analysis of heterogeneity revealed that brain areas with altered ReHo in the bilateral PCC/precuneus, left supplementary motor area, right superior parietal lobule (extending to the angular gyrus), left fusiform gyrus, and left lingual gyrus had significant between-study heterogeneity. The results from the heterogeneity analysis are summarized in Table 3 and Fig. 3.

**Table 3:** Regions of ReHo heterogeneity from the SDM analysis

Anatomical regions	Maximum MNI coordinate (x, y, z)	Voxels	SDM-Z value	*p* value
A. Right/Left posterior cingulate gyrus/precuneus (BA 23)	−8, − 50, 38	919	4.0	0.0001
B. Left supplementary motor area (BA 6)	− 2, −6, 52	113	3.4	0.0008
C. Right superior parietal lobule/angular gyrus (BA7)	34, −72, 52	53	3.3	0.001
D. Left fusiform gyrus (BA 19)	−40, −64, −18	54	3.2	0.001
E. Left lingual gyrus (BA 17)	0, −94, 0	17	2.8	0.003

Abbreviations: *ReHo* regional homogeneity, *MNI* Montreal Neurological Institute, *SDM* Seed-based *d* Mapping, *BA* Brodmann area

### Publication bias analysis

Publication biases were detected in the bilateral PCC (*p* = 0.011) and left parahippocampal gyrus (extending to the left hippocampus) (*p* = 0.034). No publication biases for other brain regions identified in the SDM map were observed, which were revealed by the non-significant Egger’s tests (Table 2).

### Subgroup meta-analysis

The subgroup meta-analysis of studies using 3.0 T MRI scanners (*N* = 9) demonstrated that patients with aMCI compared to healthy controls exhibited increased ReHo in the bilateral lingual gyri (extending to the left middle occipital gyrus and calcarine fissure/surrounding cortex), left parahippocampal gyrus (extending to the left hippocampus), right paracentral lobule (extending to the right supplementary motor area), right postcentral gyrus, and right lingual gyrus, and decreased ReHo in the right angular gyrus (extending to the right superior parietal lobule), left fusiform gyrus (extending to left the inferior temporal gyrus), right DLPFC, left insula, and left PCC (extending to the left precuneus). The jackknife sensitivity analyses revealed that regions with ReHo alterations in the right postcentral gyrus, and right lingual gyrus, and left PCC (extending to the left precuneus) were less robust as they were only replicable in 4, 5, and 6 out of 9 combinations of datasets, respectively. Other regions showed relative robustness as they were replicable in at least 7 out of 9 combinations of datasets. The heterogeneity analysis showed significant statistical heterogeneity of ReHo in the right angular gyrus (extending to the right superior parietal lobule) and left PCC (extending to the left precuneus). No significant statistical heterogeneity of ReHo in other regions reported above was observed. Egger’s tests revealed no publication biases in the regions reported (all *p* > 0.05). The detailed results are presented in Additional file 2.

The subgroup meta-analysis of studies that matched for sex between patients with aMCI and healthy controls (*N* = 10) showed that patients with aMCI relative to healthy controls exhibited increased ReHo in the right paracentral lobule (extending to the supplementary motor area and postcentral gyrus), right lingual gyrus (extending to the bilateral calcarine fissure/surrounding cortex), and left parahippocampal gyrus (extending to the left hippocampus), and decreased ReHo in the bilateral PCC (extending to the precuneus), right middle temporal gyrus, right DLPFC, and right angular gyrus (extending to the right inferior parietal lobule). The jackknife sensitivity analyses revealed that regions with ReHo alterations in the in the right paracentral lobule (extending to the supplementary motor area and postcentral gyrus), right lingual gyrus (extending to the bilateral calcarine fissure/surrounding cortex), left parahippocampal gyrus (extending to the left hippocampus), bilateral PCC (extending to the precuneus), and right angular gyrus (extending to the right inferior parietal lobule) were replicable in 9 combinations out of 10 datasets. Right middle temporal gyrus and right dorsolateral prefrontal cortex (BA 9) were replicable in 7 and 8 combinations out of 10 datasets, respectively. The heterogeneity analysis showed significant statistical heterogeneity of ReHo in the left parahippocampal gyrus (extending to the left hippocampus), bilateral PCC (extending to the precuneus), and right angular gyrus (extending to the right inferior parietal lobule). No significant statistical heterogeneity of ReHo in other regions reported above was observed. Egger’s tests revealed publication biases in the right lingual gyrus (extending to the bilateral calcarine fissure/surrounding cortex) and bilateral PCC (extending to the precuneus) (*p* < 0.05). The detailed results are presented in Additional file 3.

### Meta-regression analysis

**Fig. 4:**
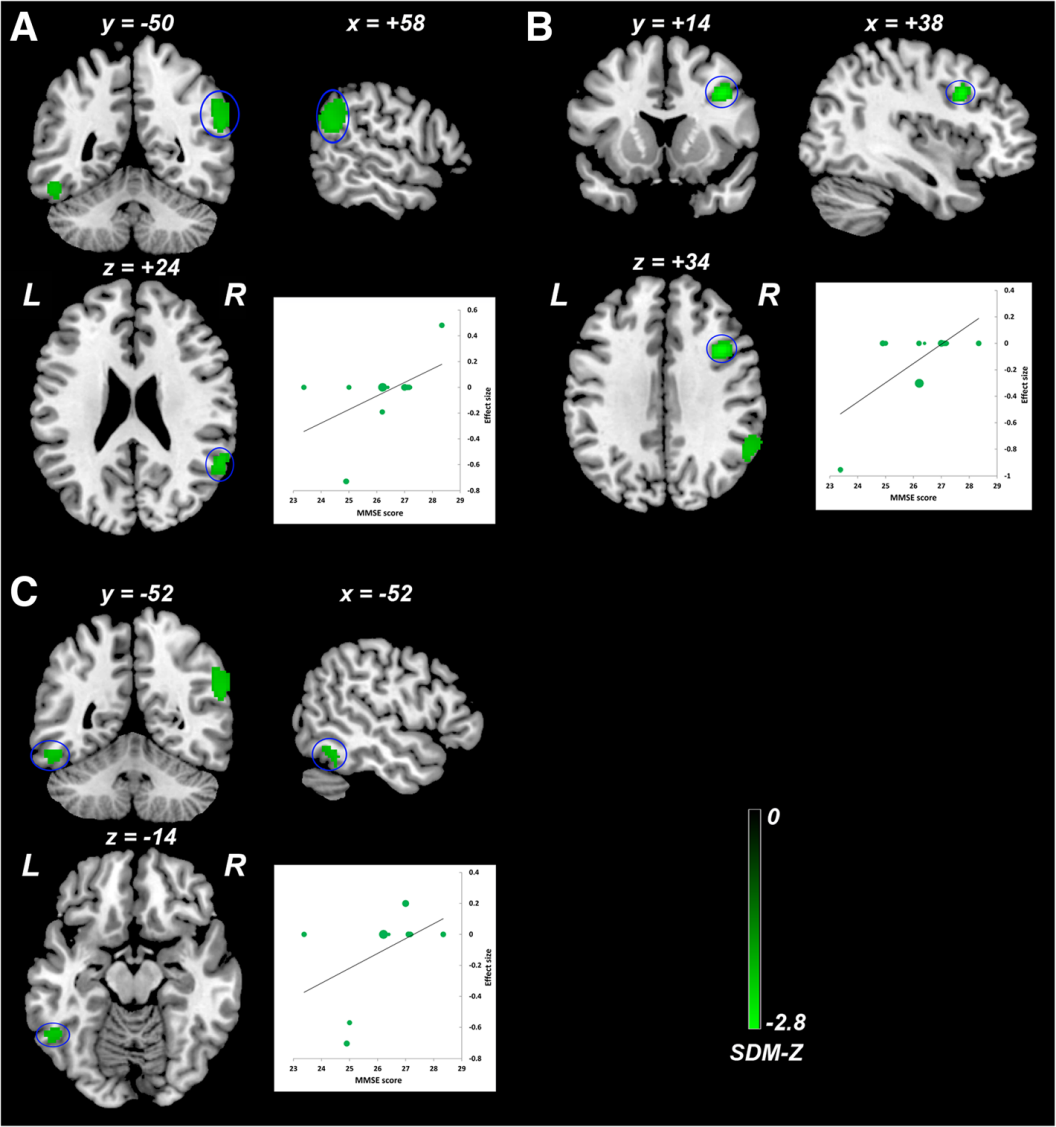
Meta-regression analysis of ReHo alterations against the mean MMSE scores across studies. Lower mean MMSE score in the aMCI sample was associated with lower ReHo in the right angular gyrus/supramarginal gyrus (**a**), right DLPFC (**b**), and left inferior temporal gyrus (**c**). The color bar indicates the maximum and minimum SDM-Z values. Each study is labeled as a dot, with larger dots symbolizing larger sample sizes. Abbreviations: ReHo, regional homogeneity; MMSE, Mini-Mental State Examination; aMCI, amnestic mild cognitive impairment; DLPFC, dorsolateral prefrontal cortex; L, left; R, right; SDM, Seed-based *d* Mapping

**Fig. 5:**
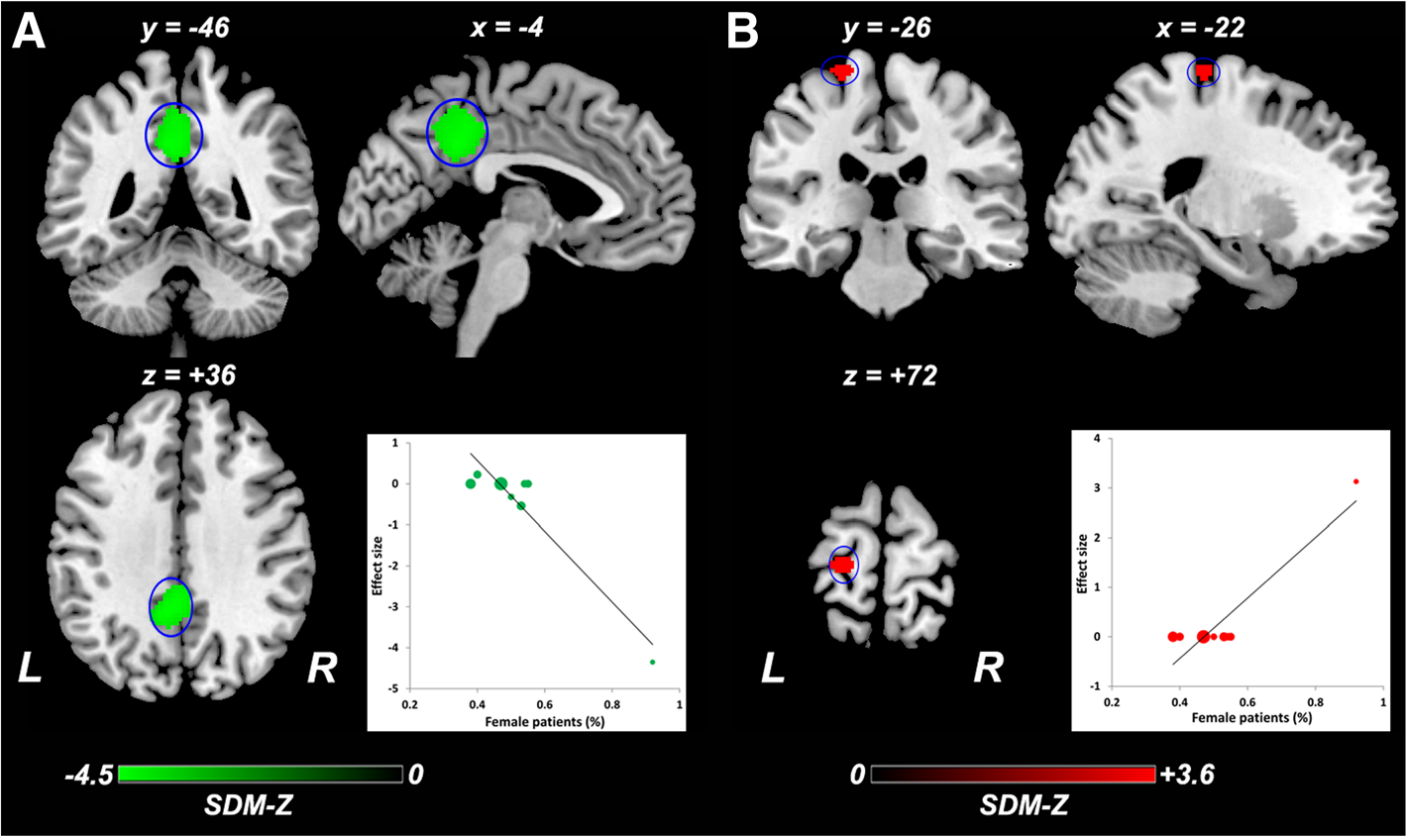
Meta-regression analysis of ReHo alterations against the female ratio across studies. More females in the aMCI samples (female ratio) were related to lower ReHo in the left PCC/precuneus (**a**) and higher ReHo in the left pre/postcentral gyrus (**b**). The color bar indicates the maximum and minimum SDM-Z values. Each study is labeled as a dot, with larger dots symbolizing larger sample sizes. Abbreviations: ReHo, regional homogeneity; aMCI, amnestic mild cognitive impairment; L, left; R, right; SDM, Seed-based *d* Mapping

**Fig. 6:**
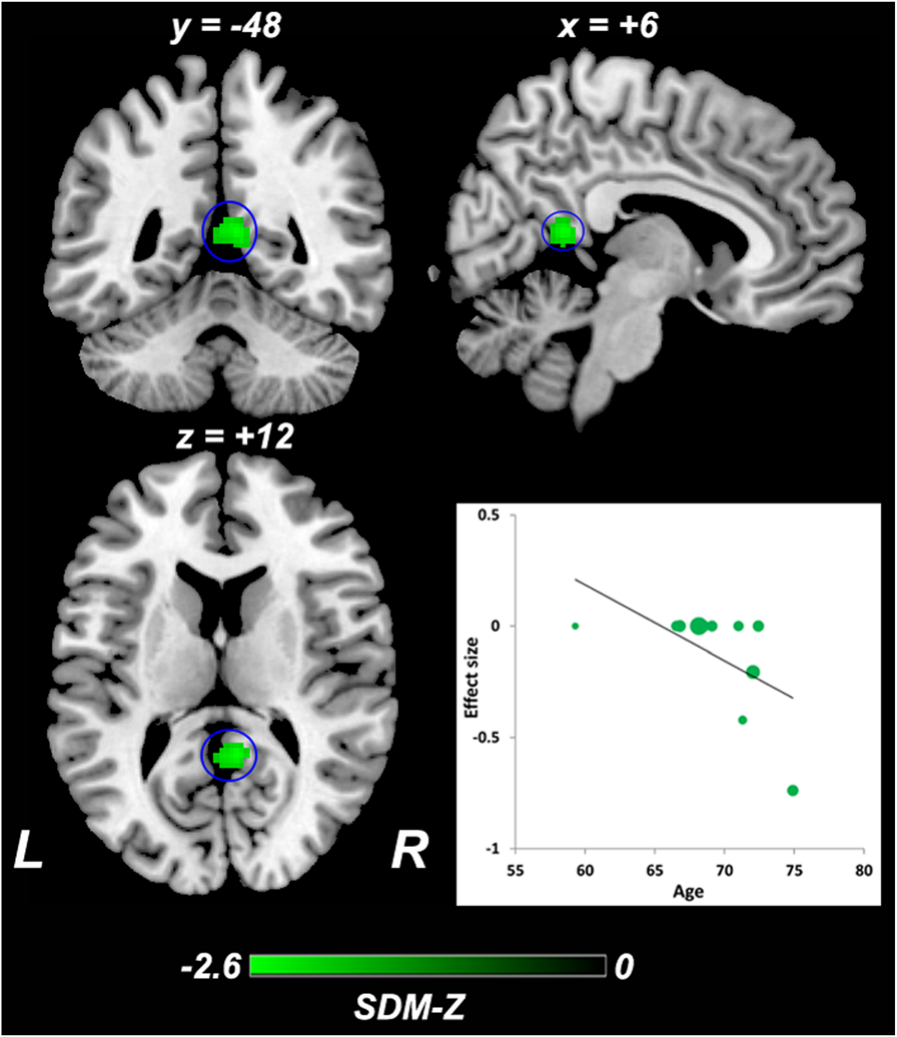
Meta-regression analysis of ReHo alterations against the mean age across studies. Mean age in the aMCI sample was negatively correlated with ReHo in the right precuneus. The color bar indicates the maximum and minimum SDM-Z values. Each study is labeled as a dot, with larger dots symbolizing larger sample sizes. Abbreviations: ReHo, regional homogeneity; aMCI, amnestic mild cognitive impairment; L, left; R, right; SDM, Seed-based *d* Mapping

**Fig. 7:**
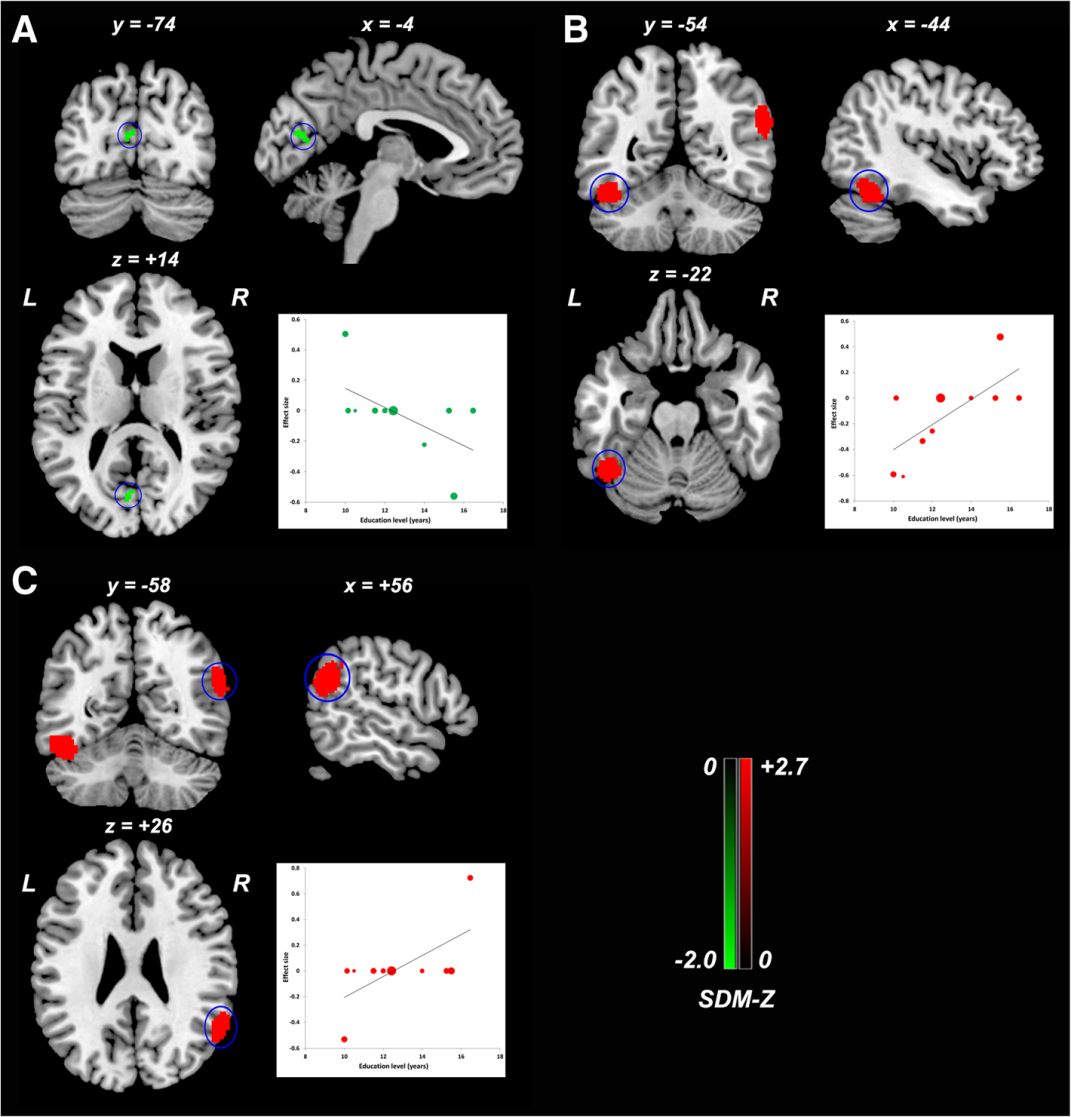
Meta-regression analysis of ReHo alterations against the educational level across studies. Higher educational level in the aMCI sample was associated with lower ReHo in the left calcarine fissure/surrounding cortex (**a**), and higher ReHo in left fusiform gyrus/inferior temporal gyrus (**b**) and right angular gyrus/superior temporal gyrus (**c**). The color bar indicates the maximum and minimum SDM-Z values. Each study is labeled as a dot, with larger dots symbolizing larger sample sizes. Abbreviations: ReHo, regional homogeneity; aMCI, amnestic mild cognitive impairment; L, left; R, right; SDM, Seed-based *d* Mapping

The meta-regression analysis showed that severer general cognitive function impairment revealed by lower MMSE score in the aMCI samples was associated with less ReHo in the right angular gyrus/supramarginal gyrus (Fig. 4a), right DLPFC (Fig. 4b), and left inferior temporal gyrus (Fig. 4c). More females in the aMCI samples (female ratio) were related to less ReHo in the left PCC/precuneus (Fig. 5a) and greater ReHo in the left pre/postcentral gyrus (Fig. 5b). Mean age in the patient samples was negatively correlated with ReHo in the right precuneus (Fig. 6). Higher educational level in the aMCI sample was associated with lower ReHo in the left calcarine fissure/surrounding cortex (Fig. 7a) and higher ReHo in left fusiform gyrus/inferior temporal gyrus (Fig. 7b) and right angular gyrus/superior temporal gyrus (Fig. 7c). Table 4 summarizes the results of the meta-regression analyses.

**Table 4:** Meta-regression analyses

Anatomical label	Peak MNI coordinate (x, y, z)	Voxels	SDM-Z value	*p* value
Effect of general cognitive function measured with MMSE score				
A. Right angular gyrus/supramarginal gyrus (BAs 39, 40, and 22)	58, −50, 24	371	−2.3	0.0001
B. Right dorsolateral prefrontal cortex (BA 9)	38, 14, 34	139	−2.8	0.00004
C. Left inferior temporal gyrus (BA 20)	−52, −52, −14	97	−2.2	0.0002
Effect of gender distribution				
A. Left posterior cingulate gyrus/precuneus (BA 23)	−4, −46, 36	689	− 4.5	0.00002
B. Left pre/postcentral gyrus (BAs 6 and 4)	−22, −26, 72	50	3.6	0.0002
Effect of age				
Right precuneus (BA 29)	6, −48, 12	121	−2.6	0.00004
Effect of education level				
A. Left calcarine fissure/surrounding cortex (BAs 17 and 18)	−4, −74, 14	25	−2.0	0.0002
B. Left fusiform gyrus/inferior temporal gyrus (BAs 37 and 20)	−44, −54, −22	321	2.7	0.00003
C. Right angular gyrus/superior temporal gyrus (BAs 39, 40 and 22)	56, −58, 26	315	2.6	0.00004

Abbreviations: *MMSE* Mini-Mental State Examination, *MNI* Montreal Neurological Institute, *SDM* Seed-based *d* Mapping, *BA* Brodmann Area

## Discussion

To the best of our knowledge, this is the first study to employ a meta-analytic approach to demonstrate ReHo differences between patients with aMCI and healthy controls. This meta-analysis identified significant brain ReHo alterations in patients with aMCI relative to healthy controls, mainly within the default mode network (DMN) (bilateral PCC, right angular gyrus, bilateral middle temporal gyri, and left parahippocampal gyrus/hippocampus), executive control network (right superior parietal lobule and DLPFC), visual network (right lingual gyrus and left middle occipital gyrus), and sensorimotor network (right paracentral lobule/supplementary motor area, right postcentral gyrus and left posterior insula). It should be noted that significant heterogeneity of ReHo alterations in the bilateral PCC, left parahippocampal gyrus/hippocampus, and right superior parietal lobule/angular gyrus was observed in the meta-analysis. The subgroup meta-analyses revealed that MR field-strength and sex partly affected the overall results. Further meta-regression analyses indicated that some confounding factors, such as general cognitive function, gender distribution, age, and education level could partially contribute to the heterogeneity across studies.

This voxel-wise meta-analysis identified significant decreases of ReHo in the bilateral PCC, right angular gyrus, and bilateral middle temporal gyri and increases of ReHo in the left parahippocampal gyrus/hippocampus in patients with aMCI relative to healthy controls. These regions of ReHo alterations are critical hubs of the DMN [33]. The DMN, which is implicated in self-referential and memory processing, is the most studied brain cognitive network in aMCI and AD because it is preferentially susceptible to neurodegeneration and involves early in the AD pathophysiology [33, 34]. Convergent evidence suggests that remote functional connectivity in the DMN in AD and aMCI is reorganized, which correlates with cognitive impairments [33–35]. Our findings in alterations of local functional connectivity within the DMN yielded complimentary insights, which contribute to the modeling of brain functional connectomes of aMCI. Functional connectivity alterations in the DMN have been reported as a predictor of conversion from aMCI to AD [36, 37]. Even more importantly, the DMN has been gaining attention as a potential target of non-invasive brain stimulation for AD [33]. Both decreases and increases of ReHo were found in the DMN. This coexistence of functional deficits and compensation in aMCI was also suggested by previous studies [38–43]. The parahippocampal gyrus and hippocampus located in the medial temporal lobe, where the hallmark of pathological changes in AD, are critical structures responsible for mnestic functions. Increased ReHo in the parahippocampal gyrus/hippocampus observed in this meta-analysis may serve to compensate for its neurodegeneration underlying memory impairments in patients with aMCI. It has been proposed that a U-shaped curve of the medial temporal lobe activation takes place early in the course of prodromal AD [44]. However, to what extent of the compensation of local functional connectivity in the aging-aMCI-AD continuum, further longitudinal studies are warranted to calcify this question.

Recent studies have shown that aMCI is associated with an aberrant topological organization of large-scale functional brain networks beyond the DMN [41, 45, 46]. Apart from the regions within the DMN, our meta-analysis identified other regions of ReHo alterations in patients with aMCI, mainly involving the executive control network, visual network, and sensorimotor network. Regions of decreased ReHo in right DLPFC and superior parietal lobule are the components of the executive control network, which is implicated in initiating and modulating cognitive control. Despite the cardinal deficits in memory, patients with aMCI also exhibited impairments in executive functioning [47–50]. The decreased ReHo in the executive control network suggested the reduced executive control ability in patients with MCI. Additionally, our meta-analysis identified increased ReHo in the right lingual gyrus and left middle occipital gyrus. These regions are important nodes of the visual network, which is crucial for the visual information processing relating to visual cognition. Previous functional MRI studies suggested that the visual network in MCI was less affected than that in AD [41]. Impairment of visual cognition is pronounced in AD but not in aMCI [51, 52]. Thus, increased ReHo in the visual network may be interpreted as a compensatory process in patients with aMCI. We also observed consistent ReHo alterations within the sensorimotor network. Pathological or structural changes in the sensorimotor cortices were found to be least affected in aMCI and relatively preserved in AD [53–55]. Increased ReHo within the sensorimotor network might be compensated to disruptions of other functional networks.

Of note is that significant heterogeneity of ReHo alterations in the bilateral PCC, left parahippocampal gyrus/hippocampus, and right superior parietal lobule/angular gyrus was observed in the meta-analysis. The subgroup meta-analyses indicated that MR field-strength and sex had confounding effects on the overall results of ReHo alterations. Exploratory meta-regression analyses revealed that this heterogeneity was attributed to some confounding factors, such as general cognitive function, gender distribution, age, and education level. Such moderators on brain structure or function have been well documented [39, 56–62]. Of interest, we observed significant association between ReHo alternates in the left PCC/precuneus and left pre/postcentral gyrus and female gender. Sex differences in brain ReHo [63], resting-state functional connectivity [64], and resting-state networks [65] in healthy subjects have been described. Although there are no statistically significant sex differences in the prevalence or incidence of aMCI [66]; however, epidemiologic studies indicate that women patients with MCI have greater longitudinal rates of cognitive and functional progression than men [67]. In postmortem investigations, women showed more extensive senile plaques deposition throughout the brain than men at early neurofibrillary tangle stages [68]. Variability in brain ReHo in patients with aMCI due to sex differences is not well understood, which should be carefully studied in future studies. Although analyses of patient characteristics might help explain the heterogeneity observed across studies, they were far from sufficient. Other potential confounding factors, such as pathological heterogeneity underlying this disorder, genetic status, neuropsychological characteristics, administered medications, acquisition parameters of MRI, and preprocessing and analytic approaches, might also contribute to the heterogeneity. Unfortunately, it was not possible to conduct separate subgroup and meta-regression analyses to systemically examine these moderating effects because of insufficient information available from the original studies, but these confounding effects merit future investigations.

Several limitations in the present study should be noted. First, we observed some publication biases in the bilateral PCC and left parahippocampal gyrus/hippocampus. In order to increase the quality of the meta-analysis, only peer-reviewed articles using a whole-brain analysis published in English were included in this meta-analysis. Thus, studies published in other languages, unpublished materials, or studies performing with ROI and two-dimensional analyses were excluded. In addition, of the 11 datasets included in the meta-analysis, eight were from China (280 patients with aMCI), two from the Alzheimer’s Disease Neuroimaging Initiative (ADNI) database (64 patients with aMCI), and one from Korea (34 patients with aMCI). Due to these factors, publication bias might be not avoidable. Hence, an additional research need exists for future comprehensively pooling big neuroimaging data from worldwide populations. Second, although patients with aMCI are heterogeneous in etiology, the ReHo findings in our meta-analysis may represent a common pattern of functional changes underlying aMCI. However, limited by the cross-sectional design of the original studies included, further clinical outcomes of patients with aMCI that convert to AD or remain stable or improve can vary considerably. Our study could not disentangle the ReHo differences between them. In addition, the cross-sectional design of the studies could not elucidate the causality of the alterations in ReHo and aMCI. Further longitudinal follow-up designed studies are highly warranted to clarify these issues. Third, as already mentioned above, many confounding factors might influence ReHo alterations on aMCI. However, not all studies made enough adjustment for these confounders. To reduce heterogeneity and provide more conclusive findings, larger and better designed studies in homogenous patients are needed. Finally, the coordinate-based meta-analysis approaches are important for identifying the anatomical convergence of neuroimaging studies. However, they mainly relied on published coordinates and their effect sizes, not on original imaging data, which may bias the results.

## Conclusions

This voxel-wise meta-analysis provides provisional evidence that aMCI is associated with abnormal ReHo within the DMN, executive control network, visual network, and sensorimotor network. The decreased and increased local connectivity in aMCI suggest coexistence of functional deficits and compensation in these networks. These findings contribute to the modeling of brain functional connectomes and to a better understanding of the neural substrates underlying cognitive deficits associated with aMCI. This meta-analysis also provides quantitative evidence that general cognitive function, gender distribution, age, and education level are confounding factors that lead to heterogeneity in the results and merit future investigations.

## Additional files

Additional file 1: Criteria for objective assessment of methodological quality of individual studies. Notes: A maximum score of 20 for each study, allocated as per the criteria specified above. (DOCX 19 kb)

Additional file 2: Subgroup meta-analysis of studies using 3.0 T MRI scanners (*N* = 9). Abbreviations: N, number of datasets; ReHo, Regional Homogeneity; MNI, Montreal Neurological Institute; SDM, Seed-based *d* Mapping; BA, Brodmann area. (DOCX 25 kb)

Additional file 3 Subgroup meta-analysis of studies that matched for sex (*N* = 10). Abbreviations: N, number of datasets; ReHo, Regional Homogeneity; MNI, Montreal Neurological Institute; SDM, Seed-based *d* Mapping; BA, Brodmann area. (DOCX 24 kb)

## Abbreviations

AD: Alzheimer’s dementia
ADNI: Alzheimer’s Disease Neuroimaging Initiative
AFNI: Analysis of Functional NeuroImages
aMCI: Amnestic mild cognitive impairment
BA: Brodmann area
BRAT: Brainnetome fMRI toolkit
CI: Confidence interval
DLPFC: Dorsolateral prefrontal cortex
DMN: Default mode network
DPARSF: Data Processing Assistant for Resting-State fMRI software
DPARSFA: DAPRSF Advanced edition
FWHM: Full-width at half maximum
HC: Healthy control
MMSE: Mini-Mental State Examination
MNI: Montreal Neurological Institute
MOOSE: Meta-analysis Of Observational Studies in Epidemiology
NA: Not Available
PCC: Posterior cingulate cortex
ReHo: Regional homogeneity
REST: The Resting-State fMRI Data Analysis Toolkit
ROI: Region of interest
rs-fMRI: Resting-state functional magnetic resonance imaging
SD: Standard Deviation
SDM: Seed-based *d* Mapping
SMD: Standardized mean difference
SPM: Statistical Parametric Mapping
