# Correction to: Alterations of brain local functional connectivity in amnestic mild cognitive impairment

**DOI:** 10.1186/s40035-018-0140-x

**Published:** 2018-12-24

**Authors:** Dan Zheng, Wei Xia, Zhong Quan Yi, Pan Wen Zhao, Jian Guo Zhong, Hai Cun Shi, Hua Liang Li, Zhen Yu Dai, Ping Lei Pan

**Affiliations:** 1 School of Nursing, Jiangsu Vocational College of Medicine, Yancheng, People’s Republic of China; 2 Department of Neurology, Affiliated Yancheng Hospital, School of Medicine, Southeast University, West Xindu Road 2#, Yancheng, Jiangsu Province 224001 People’s Republic of China; 3 Department of Central Laboratory, Affiliated Yancheng Hospital, School of Medicine, Southeast University, West Xindu Road 2#, Yancheng, Jiangsu Province 224001 People’s Republic of China; 4 Department of Radiology, Affiliated Yancheng Hospital, School of Medicine, Southeast University, West Xindu Road 2#, Yancheng, Jiangsu Province 224001 People’s Republic of China

**Correction to: Transl Neurodegener**

**https://doi.org/10.1186/s40035-018-0134-8**

In the original publication of this article [1] surname of the first author is wrong. The correct name of the first author should be Dan Zheng. The original publication has been corrected.
