# Supplementary material for: Alterations of brain local functional connectivity in amnestic mild cognitive impairment
Source: Transl Neurodegener. 2018 Nov 7;7:26. doi: 10.1186/s40035-018-0134-8 (PMC6220503; doi:10.1186/s40035-018-0134-8)
Supplement: Supplementary file 1 — Criteria for objective assessment of methodological quality of individual studies. Notes: A maximum score of 20 for each study, allocated as per the criteria specified above. (DOCX 19 kb) [file 40035_2018_134_MOESM1_ESM.docx]

**Additional file 1. Criteria for objective assessment of methodological quality of individual studies**

| **Category 1: Sample characteristics (10)** |
| --- |
| 1. Patients were evaluated with specific standardized diagnostic criteria (1) |
| 1. Important demographic data (age, gender, and education) were reported with mean (or median) and standard deviations (or range)) (3) |
| 1. Healthy comparison subjects were evaluated to exclude psychiatric and medical illnesses and demographic data was reported (1) |
| 1. Important clinical variables (e.g. MMSE score) were reported with mean (or median) and standard deviations (or range)) (3) |
| 1. Sample size per group > 10 (2) |
| **Category 2: Methodology and reporting (10)** |
| 1. Whole brain analysis was automated with no a-priori regional selection (3) |
| 1. Magnet strength at least 1.5T (1) |
| 1. At least 5 minutes of resting state acquisition (1) |
| 1. Whole brain coverage of resting scans (1) |
| 1. The acquisition and preprocessing techniques were clearly described so that they could be reproduced (1) |
| 1. Coordinates reported in a standard space (1) |
| 1. Significant results are reported after correction for multiple testing using a standard statistical procedure (FDR, FWE or permutation-based methods) (1) |
| 1. Conclusions were consistent with the results obtained and the limitations were discussed (1) |

**Notes:** A maximum score of 20 for each study, allocated as per the criteria specified above.
