# Supplementary material for: Alterations of brain local functional connectivity in amnestic mild cognitive impairment
Source: Transl Neurodegener. 2018 Nov 7;7:26. doi: 10.1186/s40035-018-0134-8 (PMC6220503; doi:10.1186/s40035-018-0134-8)
Supplement: Supplementary file 2 — Subgroup meta-analysis of studies using 3.0 T MRI scanners (N = 9). Abbreviations: N, number of datasets; ReHo, Regional Homogeneity; MNI, Montreal Neurological Institute; SDM, Seed-based d Mapping; BA, Brodmann area. (DOCX 25 kb) [file 40035_2018_134_MOESM2_ESM.docx]

**Additional file 2. Subgroup meta-analysis of studies using 3.0T MRI scanners (N = 9)**

|  | Anatomical label | Peak MNI coordinate  (x, y, z) | Voxels | SDM-Z value | p value  (SDM) | Heterogeneity | Sensitivity analysis | p value  (Egger's test) |
| --- | --- | --- | --- | --- | --- | --- | --- | --- |
| Increased ReHo | Bilateral lingual gyri/Left middle occipital gyrus/calcarine fissure/surrounding cortex (BAs 17 and 18) | 8, -90, -2 | 1595 | 1.7 | 0.00005 | No | 8/9 | 0.3 |
|  | Left parahippocampal gyrus/hippocampus (BAs 28 and 35) | -22, -20, -22 | 238 | 1.6 | 0.0001 | No | 8/9 | 0.06 |
|  | Right paracentral lobule/supplementary motor area (BAs 4 and 6) | 10, -32, 54 | 314 | 1.5 | 0.0002 | No | 7/9 | 0.5 |
|  | Right postcentral gyrus (BA 3) | 34, -28, 44 | 47 | 1.3 | 0.0009 | No | 4/9 | 0.7 |
|  | Right lingual gyrus (BA 18) | 4, -70, -6 | 24 | 1.1 | 0.003 | No | 5/9 | 0.8 |
| Decreased ReHo | Right angular gyrus/superior parietal lobule (BAs 7 and 39) | 38, -64, 48 | 543 | -1.9 | 0.0003 | Yes | 9/9 | 0.9 |
|  | Left fusiform gyrus/inferior temporal gyrus (BAs 37 and 19) | -46, -58, -18 | 490 | -2.0 | 0.0001 | No | 9/9 | 0.4 |
|  | Right dorsolateral prefrontal cortex (BA 9) | 36, 8, 34 | 159 | -2.0 | 0.0002 | No | 8/9 | 0.2 |
|  | Left insula (BA 13) | -34, -6, 6 | 47 | -1.5 | 0.003 | No | 7/9 | 0.9 |
|  | Left posterior cingulate gyrus/precuneus (BA 23) | -12, -50, 34 | 24 | -1.5 | 0.003 | Yes | 6/9 | 0.06 |

Abbreviations: ReHo, Regional Homogeneity; MNI, Montreal Neurological Institute; SDM, Seed-based *d* Mapping; BA, Brodmann area
