# Supplementary material for: Alterations of brain local functional connectivity in amnestic mild cognitive impairment
Source: Transl Neurodegener. 2018 Nov 7;7:26. doi: 10.1186/s40035-018-0134-8 (PMC6220503; doi:10.1186/s40035-018-0134-8)
Supplement: Supplementary file 3 — Subgroup meta-analysis of studies that matched for sex (N = 10). Abbreviations: N, number of datasets; ReHo, Regional Homogeneity; MNI, Montreal Neurological Institute; SDM, Seed-based d Mapping; BA, Brodmann area. (DOCX 24 kb) [file 40035_2018_134_MOESM3_ESM.docx]

**Additional file 3. Subgroup meta-analysis of studies that matched for sex (N = 10)**

|  | Anatomical label | Peak MNI coordinate  (x, y, z) | Voxels | SDM-Z value | p value  (SDM) | Heterogeneity | Sensitivity analysis | p value  (Egger's test) |
| --- | --- | --- | --- | --- | --- | --- | --- | --- |
| Increased ReHo | Right paracentral lobule/supplementary motor area/postcentral gyrus (BAs 4, 6 and 3) | 10, -30, 54 | 558 | 1.49 | 0.0004 | No | 9/10 | 0.6 |
|  | Right lingual gyrus/bilateral calcarine fissure/surrounding cortex (BAs 17 and 18) | 8, -90, -8 | 1595 | 1.7 | 0.0008 | Yes | 9/10 | 0.7 |
|  | Left parahippocampal gyrus/hippocampus (BAs 30 and 35) | -22, -20, -22 | 173 | 1.6 | 0.0003 | No | 9/10 | 0.03 |
| Decreased ReHo | Bilateral posterior cingulate gyrus/precuneus (BA 23) | 2, -48, 24 | 567 | -1.8 | 0.0005 | Yes | 9/10 | 0.02 |
|  | Right middle temporal gyrus (BA 21) | 52, 2, -22 | 429 | -1.6 | 0.0009 | No | 7/10 | 0.9 |
|  | Right dorsolateral prefrontal cortex (BA 9) | 40, 10, 36 | 159 | -2.0 | 0.0001 | No | 8/10 | 0.2 |
|  | Right angular gyrus/inferior parietal lobule (BAs 7 and 39) | 34, -44, 42 | 32 | -1.5 | 0.002 | No | 9/10 | 0.01 |

Abbreviations: ReHo, Regional Homogeneity; MNI, Montreal Neurological Institute; SDM, Seed-based *d* Mapping; BA, Brodmann area
